# Comparative Analysis of Effectiveness Between Flipped Classroom and Lecture-Based Classroom in Undergraduate Medical Education at Alfaisal University

**DOI:** 10.7759/cureus.11408

**Published:** 2020-11-09

**Authors:** Muhammad Sajid, Abdul Ahad Shaikh, Muhammad Faisal Ikram, Peter Cahusac, Ahmed Yaqinuddin, Wael AlKattan, Dileep Rohra

**Affiliations:** 1 Pathology, Alfaisal University College of Medicine, Riyadh, SAU; 2 Physiology, Alfaisal University College of Medicine, Riyadh, SAU; 3 Anatomical Sciences, Alfaisal University College of Medicine, Riyadh, SAU; 4 Pharmacology, Alfaisal University College of Medicine, Riyadh, SAU; 5 Surgery, Alfaisal University College of Medicine, Riyadh, SAU

**Keywords:** flipped classroom, neuropharmacology, undergraduate teaching strategies, saudi arabia

## Abstract

Objective

The effectiveness of the flipped classroom is currently debated due to conflicting results from different studies. It is therefore important to evaluate its usefulness each time it is applied in a new setting. Thus, this study was conducted with the objective of evaluating the effectiveness and acceptability of the flipped classroom in undergraduate medical education at the College of Medicine, Alfaisal University.

Methods

This was a quasi-experimental study consisting of development and administration of a flipped classroom with one group of students receiving the flipped classroom (FG) and the other group with the traditional lecture-based teaching (LG). We compared the pre-university enrolment Cumulative Grade Point Average (CGPA), preceding progress test results and previous semester performance for the two groups, which showed no statistical difference.

Results

Since the FG had received the video lecture while the LG had not, there was a clear statistical difference between the groups with FG showing better performance in pre-test scores. The post-test performances were marginally not statistically different between FG and LG groups.

Conclusion

Our results did not show any long-term benefit of a flipped classroom in terms of retention of knowledge as manifested by grades obtained in midterm and final examinations. It was also not received positively by the students.

## Introduction

Medical education has gone through an extensive evolutionary process. From traditional lecture-based teaching to problem-based learning (PBL), case-based learning, and team-based learning (TBL) are the modalities increasingly being employed in undergraduate medical education. The most effective approach to improve teaching efficiency is to promote active learning, which requires students to actively engage with learning materials, participate in the class, and collaborate with other classmates [1-4]. Active learning has been shown to work better as it engages the students in the learning process [1-3, 5]. It leads to more interaction between teachers and students and more frequent feedback from the faculty [5,6]. These active learning strategies also lead to more collaboration and interaction between the students and may lead to a more inclusive learning experience [1-3].

Alfaisal University was established in 2008 and has a hybrid curriculum. TBL is employed in years 1 and 2 while PBL is introduced in year 2 and continued till year 3 to supplement didactic lectures. Since its inception, the traditional on-campus classroom environment has been the predominant educational model for delivering course information at Alfaisal University. This method of instruction facilitates direct contact with fellow students and professors. Students meet at an assigned time and venue to attend in-class lectures. The PowerPoint (Microsoft Corporation, Redmond, USA) presentations for the lectures taught in class are uploaded onto an online platform, Moodle for the students to refer to if/when needed. Recently, the flipped classroom approach has received much attention in medical education [5-6]. The flipped classroom requires students to obtain background knowledge through homework prior to a face-to-face class meeting, and reserves class time for applying knowledge to solve real clinical problems through a discussion facilitated by faculty [7-9]. This is unlike the traditional lecture-based classroom, in which students attend didactic lectures where they obtain knowledge passively from the instructor. Previous studies have shown that the flipped classroom can provide students with more flexibility for self-paced learning, help to promote content retention, and promote students’ interest in learning [7-10]. However, the overall effectiveness of the flipped classroom approach in medical education is still being debated [11-13]. There is a debate in the literature that if a flipped classroom is conducted poorly and is not associated with summative evaluation, students do not do prior reading/ homework assigned to them [7-8, 11-13]. Therefore, it is important to evaluate the effectiveness of the flipped classroom each time that it is applied to a new setting. Thus, this study was conducted with the objective of evaluating the effectiveness and acceptability of the flipped classroom in undergraduate medical education at the College of Medicine, Alfaisal University.

## Materials and methods

This was a quasi-experimental study. Institutional Review Board (IRB) approval (vide IRB-20004) was obtained before the commencement of the study. We utilized and followed the tips and methods described previously in designing this study and for evaluating the effectiveness of flipped classroom [9]. Study participants included second-year Bachelor of Medicine, Bachelor of Surgery (MBBS) students during the Neuroscience Block. This block covers the basic concepts of neurophysiology, neuroanatomy, neuropathology and core concepts of psychiatry and neuropharmacology. As per the directives of the Saudi Ministry of Education, all lectures in the universities, including the College of Medicine, Alfaisal University are delivered twice, separately for males and females. We utilized this built-in division for our groupings. We allotted all female students to the flipped classroom group (FG) and all the males were allotted to the lecture-based classroom group (LG). Students were aware of their group assignments before the lectures. One of the investigators (DR) prepared a video-recorded lecture on “Opioids and Antagonists” for FG and uploaded it on Moodle one week before the actual class. The video was created using custom-designed PowerPoint animations and recorded and edited on Camtasia 2020 software (TechSmith Corporation, Michigan, USA). The said faculty also delivered the traditional lecture to LG on PowerPoint on the same topic. The video contained the learning objectives of the lecture to be discussed a week later and brief information about the topic. In order to stimulate the focused learning, several clinical vignettes were included in the video and open-ended questions were asked covering the intended learning objectives and the application of knowledge. It also included the suggested study material.

A discussion forum was created on online platform Moodle and the video was uploaded on it. The membership of the discussion forum was restricted to the FG group and was mandatory. Instructions were placed on the forum to watch the video and to post a comment in response to the video before the in-class discussion. An email was also sent to the FG group asking them to watch the video before the class and prepare for the discussion in the classroom. This ensured that the maximum number of participants watched the video before the flipped classroom session.

However, LG was not required to do any homework and a didactic lecture on the same topic covering the same learning objective was delivered in traditional fashion. Both the classes started with a pre-test consisting of seven problem-solving multiple-choice questions (MCQ). At the end of both flipped classroom as well as the lecture, the same MCQs were once again tested as a post-test. The pre-test and post-test files were uploaded on Examplify software (ExamSoft, Dallas, USA), which is computer-based exam testing software used by the Alfaisal University. In order to compare the short-term and long-term retention of knowledge between the two groups, two MCQs from the midterm and nine MCQs from the summative examination were included in analysis. The performance of the two groups in these MCQs was retrieved from the Examplify software. To strengthen evidence of content validity, the MCQs were reviewed in a multi-disciplinary meeting.

After the flipped classroom experience, students’ feedback regarding their learning experience was collected through a self-administered questionnaire. This questionnaire was validated and used as reported previously [13]. The questionnaire comprised of the rating of items on a five-point Likert scale and enquired about their flipped classroom experience and objective understanding. The questionnaire also enquired whether flipped classroom was useful for exam preparation, and its comparison to didactic teaching. Students were asked about what proportion of lectures they would prefer as flipped classrooms rather than didactic. An open-ended question about suggestions to improve the flipped classroom was also included.

Inclusion criteria

All the students who were enrolled in Neuroscience Block at College of Medicine, Alfaisal University.

Exclusion criteria

The students who were unable to sit in any component of the assessment due to any reason were excluded from the analysis.

Sample size

We were interested in a medium effect size of 0.5, equivalent to 7.5% improvement (from previous blocks data with an SD of 15%). For a two-tailed two independent groups t-test, with α = 0.05 and β = 0.1, the 2:3 allocation of sample size (due to inequality between male & female numbers) sample size was calculated to be 71 for males and 107 for females and total n = 178. The number of students enrolled in Neuroscience Block this year was 215 (males: 79; females: 136).

Data analysis

All the data was entered into Microsoft Excel (Microsoft Corporation, Redmond, USA) and statistical analysis was done using Jamovi (https://www.jamovi.org/). Parametric statistics were used for all analyses. Welch’s t-tests were used for independent group comparisons. Within the flipped classroom group, the consistency of the seven Likert scale questions concerning the positive aspects of the flipped classroom was assessed with Cronbach’s alpha. The feedback question concerning the percentage of topics that should be covered by flipped classroom ranges from 0% to 70% and was correlated with the mean of the Likert feedback questions.

## Results

The total number of registered students in the Neuroscience Block was 215 (136 females and 79 males). After excluding the students who failed to appear in pre- and/or post-test, the data from a total of 193 students (128 in FG and 65 in LG) was analyzed for scores in pre- and post-test. Some students failed to appear in the midterm and final examinations, so the analysis was done on the grades of 192 (128 FG and 64 LG) for midterm and 188 (123 FG and 65 LG) students.

Pre-university enrolment Cumulative Grade Point Average (CGPA), preceding progress test results (2018 and 2019) and previous semester performance in the preceding course (Pathogenesis of Disease [POD] Block) were used to compare LG and FG groups. Welch’s t-test for CGPA showed no statistical difference t(127) = 0.390, p = 0.697, FG mean = 3.179 and LG mean = 3.206. The progress test and POD block results were more relevant since they were obtained during students’ attendance at university. All the scores were standardized, and a mean standardized score calculated from all three assessments. There was no statistical difference between FG and LG groups, t(132) = 0.327, p = 0.744. These data suggest that the FG and LG performances were very close before the flipped classroom intervention.

**Figure 1:**
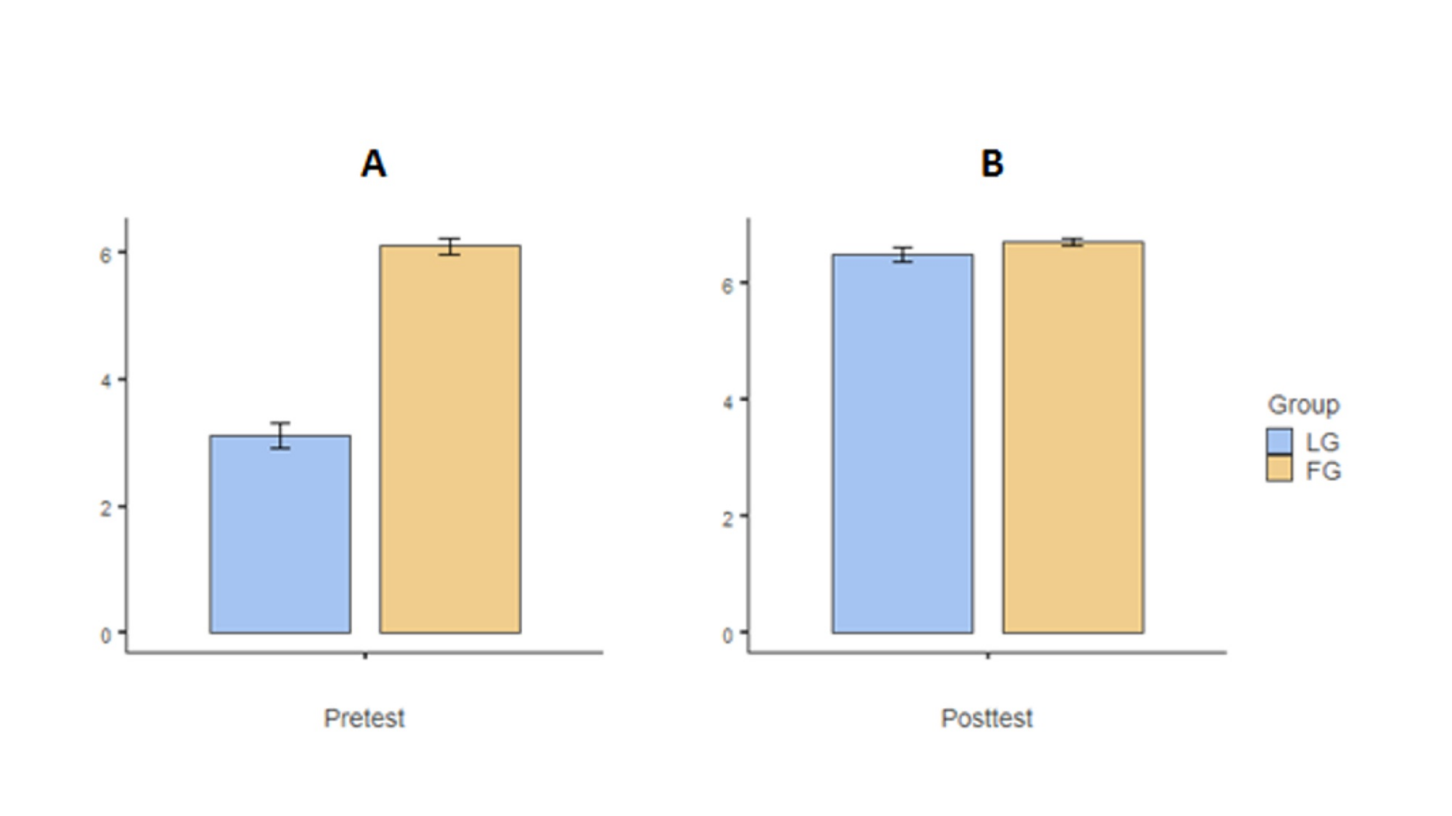
Pre-test (A) and post-test (B) results for the LG and FG. As expected, FG performed statistically better than LG in the pretest but not the posttest. Standard error bars are shown for each mean. FG: flipped classroom group; LG: lecture-based group

Pre-test scores would be expected to be different between the groups since the FG had received the video lecture while the LG had not. As shown in Figure 1, there was a clear statistical difference between the groups with FG showing better performance, t(116) = 12.769, p < 0.001, FG mean = 6.11 and LG mean = 3.11 (Figure 1A). The post-test performances were marginally not statistically different between FG and LG groups, t(101) = 1.784, p = 0.077, FG mean = 6.72 and LG mean = 6.49 (Figure 1B). A paired test comparison between pre- and post-test showed the expected improvement by the LG group, t(100) = 11.838, p < 0.001, FG mean = 0.61 and LG mean = 3.38.

**Figure 2:**
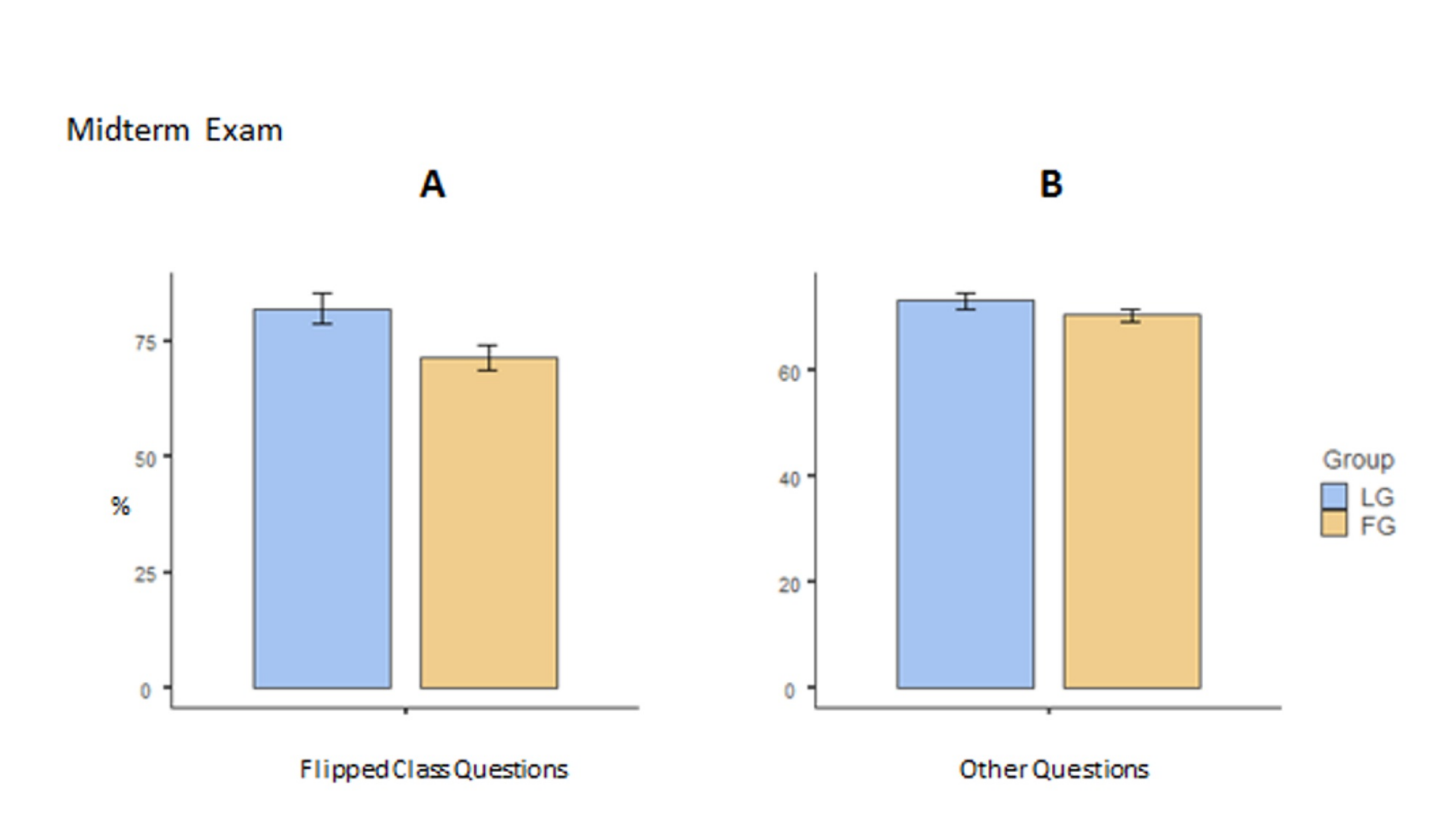
Midterm exam results for flipped class questions (A) and the remaining questions (B). LG performed statistically better than FG for the flipped class questions, while there was no difference for the remaining questions. Standard error bars are shown for each mean. FG: flipped classroom group; LG: lecture-based group

Figure 2 shows the performance of students in the midterm examination. The grades obtained by the two groups from items pertaining to the title “Opioids and Antagonists” are depicted in Figure 2A. Interestingly, the LG students fared better (statistical difference, t(145) = 2.387, p = 0.018, LG mean = 82.03 and FG mean = 71.48). As an internal control, the performance of both groups in items excluding the title of flipped classroom was also analyzed and is presented in Figure 2B. No difference in grades was observed in this analysis between the two groups.

**Figure 3:**
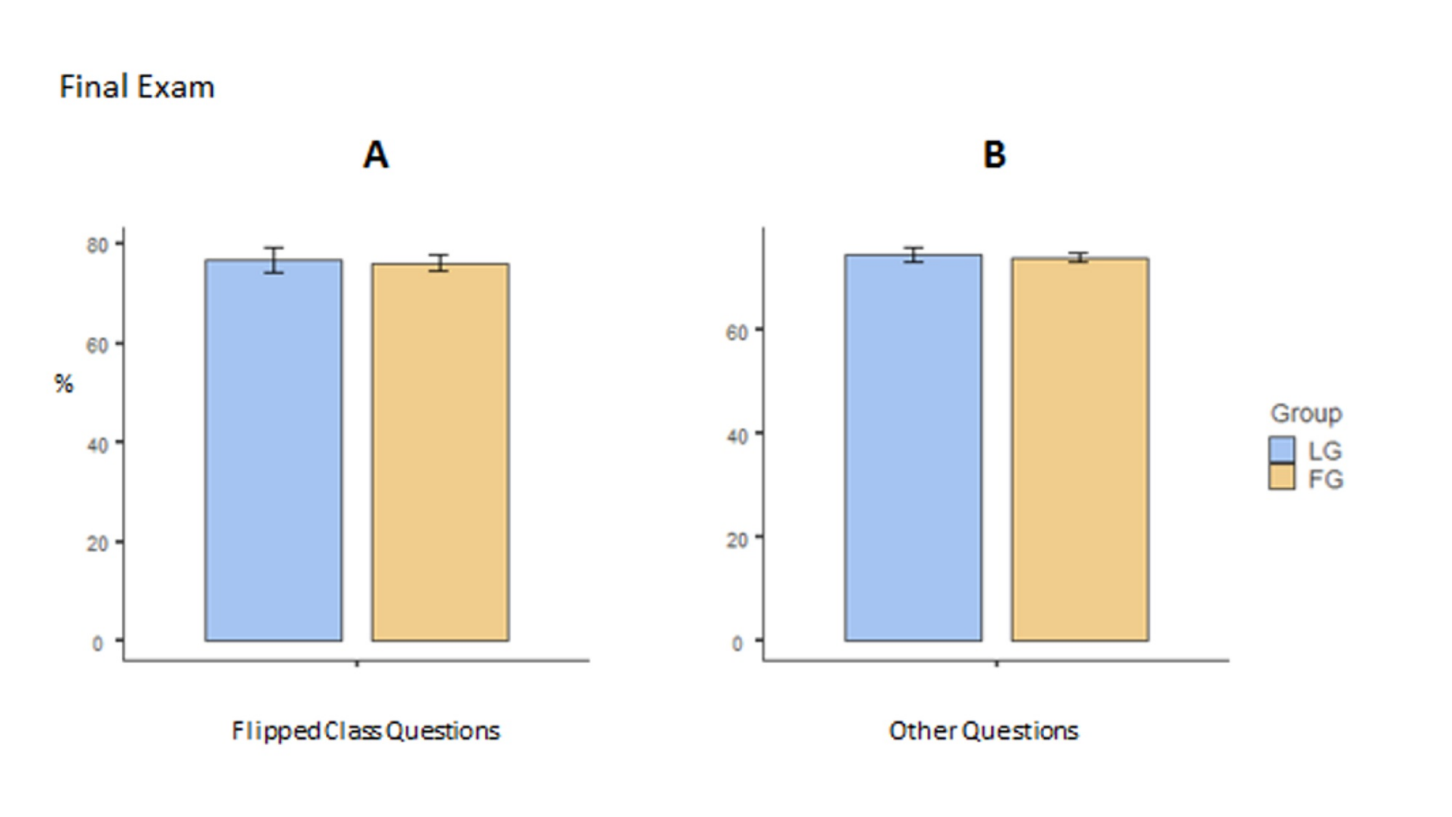
Final exam results for flipped class questions (A) and the remaining questions (B). There were no statistical differences between the groups. Standard error bars are shown for each mean. FG: flipped classroom group; LG: lecture-based group

Like midterm, an analysis was also done on the grades obtained in the final examination. Figure 3A shows the grades of students obtained in items pertaining to the flipped classroom while Figure 3B depicts the grades obtained in the whole examination excluding the items from the flipped classroom title. It is evident from this figure that there was no difference between the groups in the grades.

**Figure 4:**
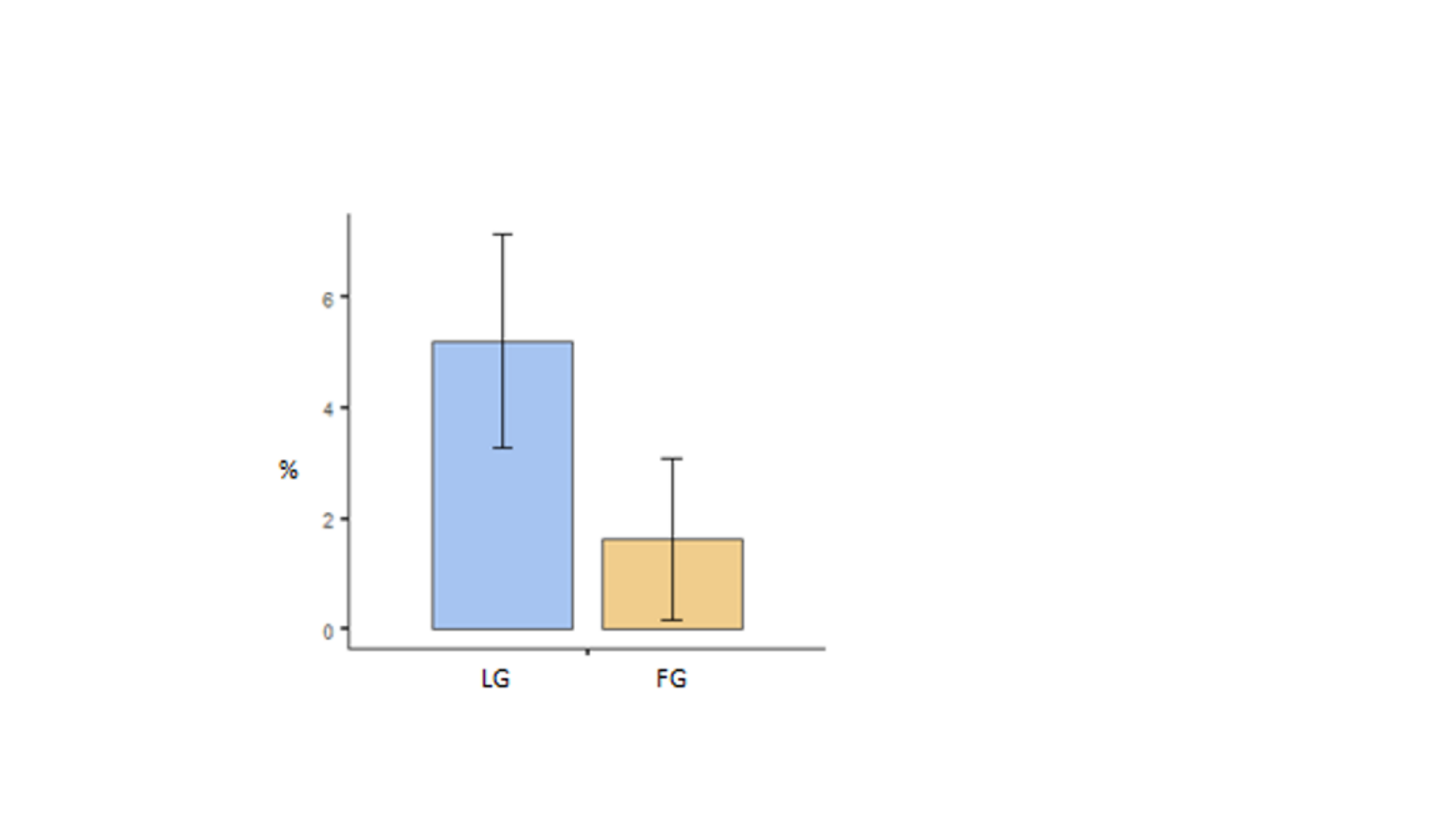
LG and FG performance represented by subtracting average % obtained in material from the intervention lecture and % obtained in material from other lectures. The data come from both mid-term and final examinations. Standard error bars are shown for each mean. FG: flipped classroom group; LG: lecture-based group

A separate analysis was also performed on the overall grades of the students. The performance for those questions concerning the particular intervention lecture was compared with performance on questions from other lectures (by simple subtraction). Mid-term and final examination results were averaged. As shown in Figure 4, LG performed slightly better than FG, t(135) = 1.476, p = 0.142, FG mean = 1.64% and LG mean = 5.21%.

A more accurate comparison can be made if the average standardized progress test and POD block results are included in an analysis of covariance (ANCOVA). The prior performance could explain some of the observed difference due to the intervention lecture, and removed from the main comparison of interest. Because there was little prior difference between the groups, this produced almost identical results to the independent samples t-test, F(1, 187) = 2.234, p = 0.137, estimated means: FG = 1.52% and LG = 5.25%. As before, these results suggest that performance was poorer in FG than in LG. In planning the study, we expected a useful effect was an improvement of 7.5% due to the intervention. Using the evidential approach with an expected improvement of 7.5% compared with no improvement, the observed data produced a likelihood ratio of 0.00018, and a log likelihood ratio of -8.6 [14, 15]. This represents extremely strong evidence in favor of no improvement versus an improvement of 7.5%. As noted earlier, the data suggest that the flipped classroom intervention decreased exam performance by around 3.7%.

Results of feedback questionnaire

**Figure 5:**
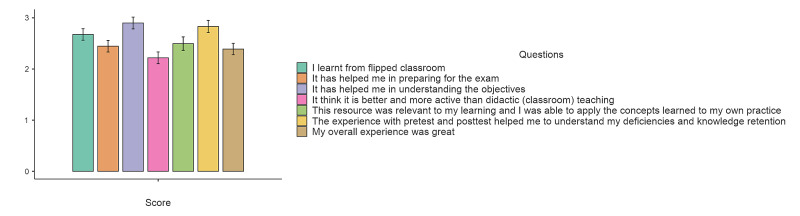
Results from the questionnaire administered to students. Results using 7-point Likert scales to the seven questions is shown. The mean response across all the questions was statistically less than the midpoint of 3.

All students in the FG were invited to fill the questionnaire, while a response from 119 students was received. Seven questions employed a five-point Likert scale (1 - Strongly disagree to 5 - strongly agree). As shown in Figure 5, all the means for the different questions were below the neutral point (3). A one-sample t-test shows that the overall mean was 0.422 less than the neutral point and statistically t(115) = 4.48, p < 0.001.

An eighth question asked what proportion of topics should be covered online through the flipped classroom. Table 1 summarizes the numbers and percentages for each option. The greatest number (77/119) suggested that none of the blocks should use this mode of teaching. The acceptability of the flipped classroom progressively decreased in response to the question related to the percentage of the block to be covered by this modality (Table 1).

**Table 1:** Response of the students regarding the percentage of course content that should be covered through flipped classroom (n=119)

Suggested % of course covered through flipped classroom	n (%)
None	77 (64.7)
20%	21 (17.6)
30%	15 (12.6)
50%	4 (3.4)
70%	2 (1.7)

Students were also asked, “What is your suggestion in improving this modality of teaching?” Just over half of the students provided an answer (62/119). The majority of responders provided negative answers (49/62) typically saying that it should not be repeated or that it was a waste of study time. Only 13/62 provided constructive comment, such as that it should not be graded. Perhaps the most positive comment was: “I felt compelled to find out as much as possible before coming to class and learning in class was very helpful in adding to my knowledge. The question guided my learning and noticed I didn’t have to review the lecture as heavily after class since class-time was like the review.”

Feedback questionnaire Cronbach’s Alpha was 0.907, representing excellent reliability.

There was a positive correlation between the mean of the seven feedback questions and the preferred proportion of topics to be covered using flipped classroom, r = 0.634, p < 0.001.

## Discussion

We designed and conducted this quasi-experimental study utilizing the tips and methods previously described to evaluate the effectiveness and acceptability of the flipped classroom in undergraduate medical education at College of Medicine, Alfaisal University compared to didactic teaching modalities such as lectures [9]. Flipped classroom teaching promotes active learning and encourages personal accountability for learning [9, 16]. In a traditional lecture, students listen to the faculty which can lure students into a ritual of memorizing and regurgitating information for their summative assessments. This can prevent them from concentrating on the clinical application of the material. Therefore, most students resort to memorizing the lecture with the objective to pass the examinations which can lead to failure on account of students to retain the knowledge [16]. 

There are also frequent complaints from faculty that students when attending lectures come without prior preparation and without completing homework or any assigned reading [16]. Previous studies have demonstrated that active learning activities in the flipped classroom increase student accountability for class preparation. It has been demonstrated that pre-class activities enhance in-class learning in previously published studies [16-21].

However, for any educational method to be considered successful, there must be evidence that student learning is enhanced. Scores in midterm and end of block examinations were considered as a surrogate for acquisition and retention of learning in this study. In this study, we found that pre-test scores which were expected to be better in the FG group since they had received the video lecture while the LG had not. Therefore, there was a clear statistical difference between the groups with FG showing better performance (Figure 1). However, this difference in performance ceased in the post-test grades between FG and LG groups. This was in concordance with previously published studies. A study involving pharmacy students in a patient self-care course found improvement in students’ scores after implementing a flipped classroom model [17]. Wong et al. showed that in comparison to a control group of students attending traditional lectures for cardiac arrhythmias, students in a flipped classroom had higher mean assessment scores [18].

Our findings do not show an increase in examination scores of students who were exposed to a flipped classroom. This finding is more in accordance with Everly and Cochran who compared two groups of students in a gastroenterology course and found no significant differences on assessment question performance between students in the traditional lecture group and the group using a flipped classroom format [22].

Student perspective and feedback

As per the objectives, this study also sought to assess the students’ views of the flipped classroom mode of learning/teaching. This was accomplished through the administration of a questionnaire to the students in the FG group. This data was not collected from the male students because they were not exposed to the flipped classroom methodology. It has been seen from previously published studies that students who are more accustomed to traditional lectures may resist the concept of the flipped classroom as they believe the onus of learning shifts to them [16, 20]. They may perceive the pre-class activities as added workload and may have anxiety regarding in-class activities coupled with the uncertainty of success [16]. “Teaching ourselves” is a phrase commonly used negatively by students and is like what findings in our survey also suggest. Students perceived pre-class activities as extra work and almost 65% of the students were not in favor of covering any block content via this modality.

Students comments regarding flipped classroom that it is not suitable for a subject like pharmacology is also in accordance with previously published studies and therefore, we believe that this modality is perhaps more suitable for teaching structure and function or clinical subjects [16, 18]. However, most published literature suggests an overall favourable response from the students [5, 6, 12-13, 17, 19, 21].

Limitations of the study

There were a few limitations to the study. First, this was a single-centre study, with the flipped-classroom approach applied in one block only. Second, the content area covered was limited to one topic of a big block and intervention was offered to the female students only. The results may have been different if the crossover methodology was applied. In view of these limitations, further studies are required to validate the findings of this study with cross over methodology.

## Conclusions

It is concluded that flipped classroom did not show any long-term benefit and was not received positively by the students. In view of these findings, we suggest that flipped classroom does not offer additional value in student learning and rather is perceived as an additional burden by the students. In view of these findings, further studies are recommended before the implementation of this teaching methodology across all courses in the University. 
